# Electrophysiological analysis of the role of novelty in the von Restorff effect

**DOI:** 10.1002/brb3.112

**Published:** 2013-02-17

**Authors:** Mauricio Rangel-Gomez, Martijn Meeter

**Affiliations:** Department of Cognitive Psychology, VU University AmsterdamThe Netherlands

**Keywords:** ERPs, N2, novelty, P3, von Restorff effect

## Abstract

Items that are distinctive with respect to their context tend to be recalled better than nondistinctive items, a finding known as the von Restorff effect. The goal of this study was to elucidate the role of novelty in this effect. In two experiments, participants performed a dual task in which they had to study words presented visually while to-be ignored sounds were played over earphones. Sounds could be either standard or novel, and words could be presented in standard or novel font. Sounds were presented either simultaneously with the words (Experiment 1) or preceding them (Experiment 2). Electrophysiological correlates of novelty processing, the N2b and P3a ERP components, were recorded while the words were studied. It was seen that cued recall was better for words presented in novel fonts than for words in a standard font (the von Restorff effect). Words presented while novel sounds were played were remembered worse (Experiment 1) or equally well (Experiment 2) than those combined with standard sounds. Words presented in novel fonts elicited enhanced N2b, P3a, P3b, and N400 components; however, none of these components were specifically larger for subsequently recalled novel-font words. A larger N2b was found for recalled than for nonrecalled words, but this effect was not specific for words presented in novel font. We hypothesized that if novelty was beneficial for memory processing, the N2–P3 complex would be more enhanced for novel words that were later recalled than for those not recalled. The data showed otherwise. This suggests that novelty processing, as indexed by the N2–P3 novelty components, is not the main cause of the von Restorff effect.

## Introduction

In the 1920s Pavlov discovered that when he wanted to demonstrate conditioning to outsiders, his dogs were often too distracted by the visitors to show a conditioned salivation response to a conditioned stimulus. Pavlov called this allocation of attentional resources toward the visitors the “what is it” response, and described it as a fundamental response to novel stimuli. He was not the first to find that novel stimuli elicit an attention shift. In fact, this response had been described already in the 1860s by Ivan Sechenov, and was later called the orienting reflex (Sokolov 1963).

One of the functions of the orienting reflex might be to support learning about the novel stimulus, and there are indeed indications that novelty is related to enhancements in memory storage. One of the strongest is the von Restorff effect, named after Hedwig von Restorff. She established in 1933 that when presented with a salient stimulus, different from the rest of a study list, people tend to have better recall (and slightly better recognition) for this stimulus than for the less distinctive ones (Von Restorff 1933). The benefits of these so-called isolate items for encoding are robust and have been replicated many times. They are already present in childhood (Cimbalo et al. 1981), and remain detectable until advanced age (Bireta et al. 2008).

The beneficial effect of distinctiveness on encoding has been postulated to occur because of extra rehearsal of the isolated items that attract more attention than nonisolated items (Rundus 1971). Recent studies have shown, however, that rehearsal is not necessary for the von Restorff effect to occur, as it is seen regardless of the position on the list in which the isolate is presented (Dunlosky et al. 2000). Other experiments have shown that perceptual salience is also not necessary for this effect, as it occurs even for items presented early in the list when no context has been established yet (Dunlosky et al. 2000; Hunt and Lamb 2001), although this last argument has recently been debated (Geraci and Manzano 2010).

What causes the von Restorff effect remains unclear. There have been accounts that have emphasized processing operating at retrieval (e.g., McDaniel et al. 2005), but many focus on processing at encoding (e.g., Fabiani and Donchin 1995). As early as the 1970s it has been proposed that the von Restorff effect is influenced by the extra attention paid to isolates, which can vary as a function of presentation time and position in a sequence of stimuli (Johansson 1970). Others have emphasized the importance of the novelty of the isolates (Kishiyama et al. 2004), consistent with theories that give novelty a key role in learning (Hasselmo et al. 1996; Meeter et al. 2004; Lisman and Grace 2005). Evidence for this take comes from electroencephalogram (EEG) studies with a focus on the N2 and P3 novelty components. We will first review these components, and then come back to their importance in understanding the von Restorff effect.

The novelty N2 has been related to perceptual novelty and is highly sensitive to learning, being strongly reduced with even a single repetition of the novel stimulus (Ferrari et al. 2010). Although many describe the novelty N2 as a marker of perceptual novelty exclusively, Daffner and colleagues (2000) propose that the novelty N2 component is a complex that depends not only on perceptual novelty, but also on the probability and significance of the stimulus.

The N2 has been divided into subcomponents. In an influential review article, Pritchard and colleagues (1991) proposed a division in three subcomponents, the N2a, N2b, and N2c. These have been reformulated recently by Folstein and Van Petten (2008), as mismatch negativity (equivalent to the N2a), anterior N2 (equivalent to the N2b), and posterior N2 (equivalent to the N2c). The N2a/mismatch negativity has a fronto-central maximum distribution and is conceptualized as an automatic response to an auditory outlier (Alho et al. 1994; Kujala et al. 2001). The N2b (also known as novelty N2) is a frontal component usually observed in the visual oddball task (Crottaz-Herbette and Menon 2006; Szucs et al. 2007), and is “semiautomatic”: It is elicited by an outlier consciously perceived, but that may be task irrelevant (Daffner et al. 2000; Tarbi et al. 2010). The N2c (centrally expressed) is associated with classification tasks (Kopp and Wessel 2010).

The P3 has been divided in two subcomponents, the P3a – or novelty P3 (fronto-central), and the P3b – or classic P3 (centro-parietal) (He et al. 2001; Goldstein et al. 2002; Polich and Criado 2006; Pegado et al. 2010). In 1975, two groups, using auditory (Squires et al. 1975) and visual (Courchesne et al. 1975) oddball paradigms, found a P3 component related to task-unrelated novel stimuli, which was more centro-frontally localized than the traditional parietal P3. Squires and colleagues (1975) called this fronto-central P3 the P3a. It has been associated with the evaluation of novel stimuli for subsequent behavioral action, and postulated to be a marker of a conscious attentional switching mechanism (Friedman et al. 2001), or distractibility (SanMiguel et al. 2010). It is enhanced by low-probability events (Squires et al. 1975), and is sensitive to physical deviance, but not to other types of deviance from context, like semantic (Arbel et al. 2010). An N2b commonly precedes the P3a component (creating the N2–P3 novelty complex). It has been argued that the P3a, or novelty P3 is dependent on the relevance of the stimulus for the task (Tarbi et al. 2010), contrary to the N2b/novelty N2. Other lines of evidence, however, indicate that the P3a can be present even with stimuli that are task irrelevant (Knight and Scabini 1998; Polich 2007).

The P3b component may index stimulus meaning and significance, more than novelty detection, and is maximal at centro-parietal as opposed to fronto-central locations (Squires et al. 1975; Ferrari et al. 2010). The N2c commonly precedes the P3b component. The P3b is enhanced for stimuli that are related to later decisions or responses (Courchesne et al. 1975). It is not a simple phenomenon either; it is modulated by stimulus probability, meaning, and relevance (Knight and Scabini 1998). Thus, the P3b might be linked to the creation or revision of a stimulus representation in working memory, and context update (Donchin 1981).

Several studies have used the novelty P3 to study the role of novelty in the von Restorff effect (Karis et al. 1984; Fabiani and Donchin 1995; Wiswede et al. 2006), although the novelty N2 has usually not been looked at. These studies have suggested that the P3 indexes novelty processing that aids encoding. Using an emotional von Restorff paradigm, Wiswede and colleagues (2006) found that the P3 component is larger for remembered than for nonremembered words, but that this effect was not exclusive for isolated words; no comparison was made for the N2 component. A P3 effect was also found by Otten and Donchin (2000), who used a paradigm in which either a to-be studied word or the background was salient. They found enhanced P3 component for correctly remembered salient words and backgrounds, as compared with those that were not remembered. Fabiani and Donchin (1995) also found that isolate words elicit higher P3 components than the nonisolates, but no comparison between correctly and erroneously recalled trials was made in this study (the N2 component was again not studied).

### Current study

This study aims to investigate the involvement of novelty in encoding. We will create a von Restorff effect by changing the font, color, and size of some of the words within a list, making these words easier to recall. This robust behavioral effect will allow us to elucidate the specifics of the processing of novel stimuli, using electrophysiological techniques. If the isolated words are recognized as novel, they may generate the N2–P3 novelty complex, with higher amplitudes for novel as compared with standard words. If, in addition, this novelty value would be one reason for better encoding of isolates in a von Restorff paradigm, we would expect a correlation between N2 and P3 magnitudes and recall performance, with higher (more positive or more negative, accordingly) novelty components for words subsequently recalled correctly than for words not recalled.

To maximize the likelihood that a von Restorff effect would reflect processes at encoding and not at retrieval, we chose to use cued recall and recognition as memory measures instead of the free recall task used in the vast majority of studies of the von Restorff effect (e.g., Karis et al. 1984; Dunlosky et al. 2000; Otten and Donchin 2000; Wiswede et al. 2006). In free recall tasks, a feature that renders a word an isolate can be used as a cue. Typically, there is a single word in a study list that is printed in a larger font or color, and participants can explicitly search their memory for the large or colored item (with, e.g., large font size acting as a cue). Fabiani and Donchin (1995) offer evidence that such a strategy is indeed used, as they found that in their free recall task physical isolates (words printed in larger font) were the last to be reported, as if participants, after attempting to retrieve the rest of the list, specifically searched their memory for this item. Such a strategy would not be possible in cued recall or recognition as each item is cued separately and thus has to be retrieved on its own (and as participants do not know which cue is associated with an isolate before retrieving the word, isolate features cannot be used to inform the search for a specific word). Here, we thus presented multiple isolates within one study list (as was done by Kishiyama et al. 2004), and tested retrieval of those isolates and of standard words with cued recall and recognition tests.

If novelty aids encoding, it may also do so when novelty is not integral to the to-be studied item, but merely co-occurs with this item. Our task therefore included to-be-ignored sounds after each word. Most of the sounds were simple and repetitive “beeps”; in between the standard sounds, novel sounds were included. We also controlled for the order of the presentation of the sounds with two separate experiments. In the first, the sounds were presented together (with a slight delay) with the words. In the second, they were presented before, so that any beneficial effect of novel sounds would occur during the whole presentation of the word. We hypothesized that if novelty affects encoding, words presented with novel sounds would also be remembered better than words that are presented with standard sounds. In summary, our study differed from previous ones by looking at the novelty N2 as well as the more commonly studied novelty P3a, by using cued recall and recognition instead of free recall as our measure of memory, and by looking at the effect of novelty co-occurring with to-be studied words, but not integral to them.

## Methods

### EEG recordings: general procedure

EEG was recorded from 128 active scalp locations using the BioSemi Active2 system (Biosemi, Amsterdam, the Netherlands). Electrodes were placed according to the radial ABC system of BioSemi. Vertical and horizontal eye movements (VEOG and HEOG) were recorded, the latter using electrodes located on the outer canthus of each eye, and the former using electrodes placed below and above the right eye. Reference electrodes were located in the right and left mastoid bones. The sampling rate was set to 512 Hz.

EEG data analysis was performed using EEGlab (Delorme and Makeig 2004) and custom-written Matlab scripts. EEG data were re-referenced to the average of the signal from the two mastoid bones electrodes, resampled to 500 Hz, and digitally filtered (0.05–40 Hz; finite impulse least-square kernel with 6-dB transition of 0.01 Hz for low-pass filter and 6-dB transition of 2 Hz for high-pass filter). The data were epoched for the different conditions (novel font, standard font, novel sound, and standard sound). Epochs included 500 msec before and 1500 msec after the stimulus. The baseline was defined as the 100 msec preceding the stimulus.

An independent components analysis (ICA) was performed on the epoched data including all conditions. Independent components accounting for blink artifacts were identified and removed from the data (Jung et al. 2000a,b; Delorme et al. 2007). The data reported are, therefore, pertaining to event-related potentials. The decision about time windows of interest and electrode locations for the analysis was based on grand average waveforms for each condition.

### Participants

Participants were volunteers recruited from the student population of the Vrije Universiteit Amsterdam. All participants gave informed consent and received either money (€9 per hour) or credits for participation. None of the participants reported any psychiatric or neurological disorders. The study was performed in agreement with the Declaration of Helsinki and approved by the ethics committee of the Vrije Universiteit Amsterdam.

Twenty volunteers participated in Experiment 1, and 16 in Experiment 2. The data from four participants of Experiment 1 were removed; two due to lost data during recording, one due to excessive noise and artifacts in the EEG data, and one due to very low performance in the memory task (recall accuracy of 0%). The final group of 16 participants in Experiment 1 was composed of seven women and nine men, with ages ranging from 18 to 28 years (mean = 22 years; SD = 3.6 years). The 16 volunteers of Experiment 2 were 10 women and 6 men, with ages ranging from 20 to 31 years (mean: 26 years; SD: 3.6 years).

### Experiment 1: procedure and stimuli

The experiment was subdivided into a study phase, a cued recall phase, and a recognition phase. During the study phase, participants were presented with a list of 80 concrete nouns, with length varying between five and 10 characters, taken from the list by Van Overschelde and colleagues (2004) and complemented with an English dictionary.

All words were shown twice, in the same order, with a break after the first block. The motivation for presenting the words twice was twofold; first, it elevated recall to a level that avoided floor effects, and second, it allowed us to have more trials per condition, which is vital for ERP analysis. Each trial started with the presentation of a fixation cross for 500 msec. Then a word was presented in the middle of a gray screen (size 21′), which remained visible for 3500 msec. Words were presented either in standard font or in novel font. Standard-font words had a font size of 17 dots, with black color and courier new as font type. Novel-font words had font size of 30 dots, a variable color (one of 10 possible colors, with each color repeated twice within the list) and variable font type (unique for each novel word within a list).

Participants were seated 80 cm away from the screen, leading to the following visual angles: Standard words, 2.5–5 degrees (depending on the length of the words), for novel words, 5.7–9.6 degrees. Novel-font words were presented in the same font and color on their two presentations. The first 10 words were always presented in standard font. Of the remaining 70, a random 20 were presented in novel fonts and the remaining 50 in standard font. Word order, and assignment to condition, were randomized anew for each participant (two novel-font words could thus follow one another, although with low likelihood).

**Figure 1:**
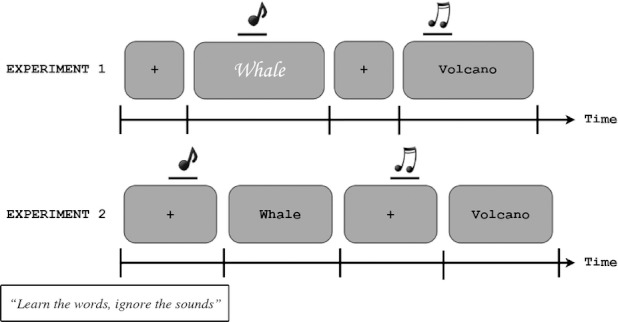
Schematic representation of the tasks used. Words were shown one by one, and presented either in standard font or in a unique, novel font (e.g., whale). Each word was combined with a sound, which could be a standard tone or a novel sound (e.g., volcano). Sounds were either during the presentation of word (Experiment 1), or preceding the presentation of a word (Experiment 2). No novel-font words appeared in Experiment 2.

During the presentation of the word, after a variable delay (from 817 to 1797 msec, mean 1344 msec, to ensure an accurate baseline for the ERP data), a sound was presented. Sounds were of two types; either a standard “beep” tone (2.2 kHz, 300 msec) presented in 58 of 80 trials, or a novel, nonfamiliar sound clip belonging to one of three different categories, namely animal, human, and mechanical sounds (previously used in Sambeth et al. 2006). The latter were presented in 22 of 80 trials. Novel sounds were presented only after the 11th word, in order to establish a context in which the standards would be recognized as occurring on most trials. After that, presentation of the sounds was random. This randomization resulted in the words and sounds conditions to be fully crossed. Thus, a novel sound could also co-occur with a novel-font word, although this rarely happened (on average on five trials per participant; see Fig. 1).

For the study phase, participants were instructed to learn the words and ignore the sounds. After the study phase, participants were asked to recall 40 of the previously learned words (a random 20 standard and all 20 novel). They were cued with the first two letters of each word and then had to complete that cue with a studied word (e.g., the “To” had to be completed to “tomato”, if that was a studied word). The cues were all presented in the same format, which was the one used for the standard-font words. After this cued recall phase, a recognition phase was presented. The recognition task included 80 words, all of them presented in standard font; 40 of those were the studied words already tested in the recall phase (20 novel and 20 standard) and 40 lures not presented before. Studied words and lures were all concrete nouns, and were picked randomly from one pool of 120 such nouns. Participants typed “z” for already presented words and “n” for not-seen-before words.

### Experiment 2: procedure and stimuli

The second experiment had the study and recall phases of Experiment 1 (but no recognition phase). Major changes with respect to the Experiment 1 were: During the study phase, words were all presented as in the standard-font condition of Experiment 1: black, 17-dot courier new. Additionally, sounds were presented before the words, instead of after.

Each trial started with the presentation of a fixation cross with duration varying from 305 to 699 msec (mean 501 msec). Then a sound was presented, while a fixation cross was still on the screen. After a variable delay (ISI from 556 to 944 msec, mean: 757), the words were presented for 2 sec. As in Experiment 1, 58 of 80 sounds were standard and 22 novel, and were presented randomly.

Instructions for participants were the same as for Experiment 1, with the difference that it was explained that sounds would occur before the words. After the study phase, participants were asked to recall all the previously learned words; again using a cued recall task (see Fig. 1).

### Data analysis

In Experiment 1, comparisons were made between novel and standard conditions for both fonts (visual novelty) and sounds (auditory novelty), based on font- and sound-stimulus-locked ERPs. Additionally, a comparison was made between words that were correctly and incorrectly recalled for the two font conditions (Wiswede et al. 2006). The recognition task was not considered for this comparison due to the small amount of trials that were not correctly recognized.

Components for the analysis were defined on a participant-by-participant basis, finding the peak or the average amplitude, for time windows obtained by visual inspection of the grand average ERPs (as in previous von Restorff studies, such as Fabiani and Donchin 1995; Wiswede et al. 2006) and in concordance with the existing literature for the electrode sites to be considered (using the same midline electrodes as Daffner et al. 2000). As the N2b component overlaps with the P2 component, a peak-to-peak methodology was used (as used elsewhere for extracting N2 effects that overlap with P2, Perrault and Picton 1984a,b). The N2b was defined as the difference between the negative peak from 170 to 200 msec, and the positive peak between 200 and 250 msec. The N2a to novel sounds was defined as the negative peak between 180 and 250 msec. The P3a component was defined as the average amplitude between 330 and 380 msec (for the fonts), and between 250 and 350 msec (for the sounds). The P3b was defined as the amplitude average between 350 and 550 msec for the sounds, and between 380 and 600 msec for the fonts.

The N2b component was computed at the Fz electrode (Folstein and Van Petten 2008), the P3a component at the Cz electrode and the P3b component at the Pz electrode (He et al. 2001; Polich and Criado 2006). These electrodes were used for the font and sound condition.

In Experiment 2, comparisons were made only between novel and standard conditions for the sounds (auditory novelty), based on sound-stimulus-locked ERPs. Components for the analysis were defined by visual inspection, resulting in the following time windows: The N2b component was defined as the negative peak between 250 and 330 msec. The P3a component was defined as the average amplitude between 330 and 430 msec; and, the P3b was defined as the amplitude average between 430 and 630 msec.

## Results

### Experiment 1

#### Behavioral results

**Figure 2:**
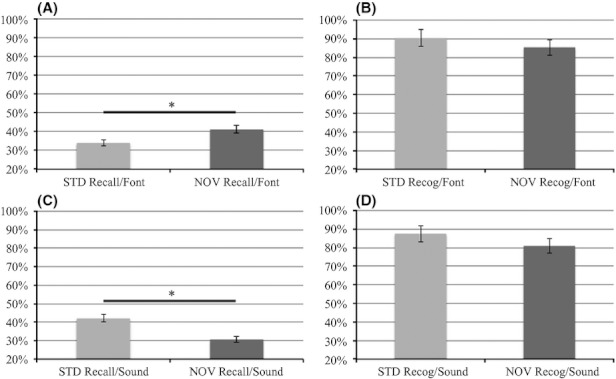
Behavioral data of Experiment 1. (A) Percentage of words recalled correctly, for novel versus standard words. (B) Recognition accuracy, for novel versus standard words. (C) Recall accuracy for novel versus standard sounds. (D) Recognition accuracy, for novel versus standard sounds. *P < 0.05. Error bars represent the 95% confidence interval.

Behavioral results are shown in Figure 2. Novel words were recalled more accurately than standard words (*t*_15_ = 2.45, *P* = 0.027). A novelty effect was also found in reaction times in the recall task. Participants were faster (*t*_15_ = 1.88, *P* = 0.078) in typing novel (mean RT = 7.7 sec, SD = 3.8 sec) than standard words (mean RT = 8.7 sec, SD = 4.4 sec). This effect was reversed for the recognition task, although this was only marginally significant (*t*_15_ = 2.05, *P* = 0.058). Moreover, words presented together with novel sounds were recalled less accurately than those presented with standard sounds (*t*_15_ = 2.98, *P* = 0.009); no difference was observed for the recognition task (*t*_15_ = 1.12, *P* = 0.28).

#### ERP analysis

**Figure 3:**
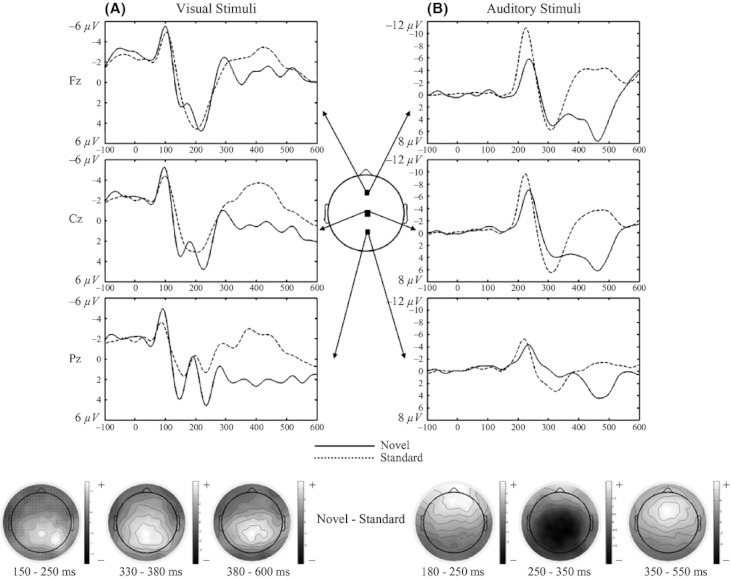
ERP plots for standard versus novel stimuli in Experiment 1. ERP plots for the comparison between novel and standard, both (A) visual and (B) auditory stimuli, for the electrodes Fz (top), Cz (middle), and Pz (bottom). The zero point corresponds to the presentation of the stimulus. A 20-Hz low-pass filter was applied for plotting purposes, but not for the analysis.

**Figure 4:**
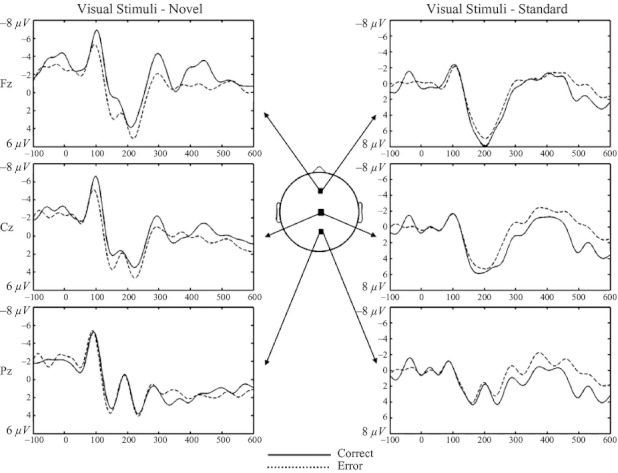
ERP plots for correct versus failed trials in Experiment 1. ERP plots for the comparison between recalled and not recalled words, for the novel and standard fonts condition. Shown are data for the electrodes Fz (top), Cz (middle), and Pz (bottom). A 20-Hz low-pass filter was applied for plotting purposes, but not for the analysis.

Figure 3 shows ERP waveforms for novel- and standard-font words, and for novel and standard sounds. Figure 4 shows ERP waveforms for correct versus incorrect trials in the novel- and standard-font conditions. For visual novelty, the data were analyzed performing a repeated measures (RM) analysis of variance (ANOVA) with novelty (novel/standard) and accuracy (correct/error) as within-subject factors. The P3a and P3b components did show such a main effect, with higher P3a amplitude for novel than for standard fonts over Cz (*F*_1,15_ = 11.09, *P* = 0.005) and higher P3b amplitude over Pz respectively (*F*_1,15_ = 7.28, *P* = 0.017). For the P3a and P3b components neither the main effect of accuracy (P3a: *F*_1,15_ = 0.006, *P* = 0.94; P3b: *F*_1,15_ = 0.30, *P* = 0.59), nor a novelty x accuracy interaction (All *F*_1,15_ < 1, *P* > 0.28) were found for the correspondent electrodes. No N2b was evident in the standard condition, so analysis was restricted to the novel-font condition. Here, no difference was found in N2b amplitude between correct and error trials over Fz (*t*_15_ = 1.32, *P* = 0.20).

For auditory novelty, only the main effect of novelty was studied, as the behavioral results made an analysis of correct versus incorrect trials on the novel sounds superfluous. The pattern was different from expected, with standard sounds eliciting a more negative N2a component over Fz (*t*_15_ = 8.19, *P* < 0.001), and a more positive P3a component over Cz, although the latter difference did not reach significance (*t*_15_ = 1.65, *P* = 0.12); the only component showing an enhancement for novel stimuli was the P3b, over Pz (*t*_15_ = 3.95, *P* = 0.001). Additional to the amplitude differences, latency differences in the N2 component were found between novel and standard sounds. This component had an earlier peak for standard sounds than for novels (*F*_1,15_ = 16.08, *P* = 0.001).

Visual inspection of the ERP waveforms showed that the differences between novel and standard fonts were not limited to the conventionally reported components. Therefore, we explored these differences in addition to the main analysis of this study. The components analyzed were the P2 and N400. The amplitude for these components was obtained either by a participant-based peak computation (P2) or by computing the average amplitude over the time window of interest (N400), according to the specific characteristics of each component.

The amplitude for the P2 (from 150 to 250 msec) component was found by computing the positive peak for each participant. The amplitude of the N400 component, as with other later components, was computed as the average amplitude of a time window of interest (380–480 msec). The N400 was analyzed over Cz as supported by the existing literature (Chwilla et al. 1995, 2007). The P2 component was analyzed for Fz, in accordance with Yuan and colleagues (2008).

The paired-samples *t*-test analysis applied to the different components showed no differences in the P2 component (*t*_15_ = 1.78, *P* = 0.09), contrary to another study using an oddball paradigm (Yuan et al. 2008). For the N400 component, the amplitude was significantly higher for novel words than for standard-font words (*t*_15_ = 4.52, *P* < 0.001). A RM ANOVA with factor correct and incorrect showed that there was no main effect of accuracy on P2 and N400 amplitude (both *F*_1,15_ < 0.6, *P* > 0.45), nor an interaction between Novelty and Accuracy (All *F*_1,15_ < 1.23, *P* > 0.28).^1^

### Experiment 2

#### Behavioral results

Recall accuracy was 30.11% (SD = 18.17) for words presented after novel sounds, and 29.74% (SD = 11.31) for words presented after standard sounds. There was no difference between these two conditions (*t*_15_ = 0.13, *P* *=* 0.89).

#### ERP analysis

**Figure 5:**
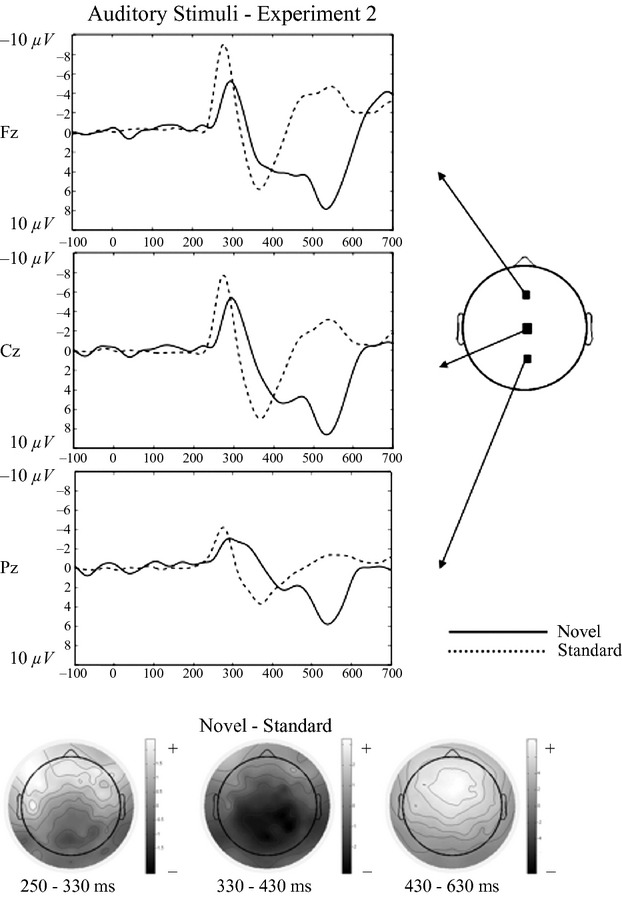
ERP plots for auditory stimuli in Experiment 2. ERP plots for the comparison between novel and standard auditory stimuli. For the electrodes Fz (top), Cz (middle), and Pz (bottom). The zero point corresponds to the presentation of the stimulus. A 20-Hz low-pass filter was applied for plotting purposes, but not for the analysis.

For Experiment 2, only one analysis was performed. We looked at differences in the ERP components between novel and standard sounds (see Fig. 5). We used the same methodology applied for the sounds in the analysis of Experiment 1. Paired-samples *t*-tests were applied for each component, comparing novel versus standard conditions. Similar to Experiment 1, standard sounds elicited a larger amplitude N2b component over Fz (*t*_15_ = 4.67, *P* < 0.001), and a larger amplitude P3a component over Cz, which was now significant (*t*_15_ = 3.03, *P* = 0.008); the only component showing an enhancement for novel stimuli was the P3b, over Pz (*t*_15_ = 4.98, *P* < 0.001).

## Discussion

This study aimed to assess the role of novelty in the von Restorff effect, and thus to investigate whether there is a beneficial effect of novelty on memory encoding. We used a task with two types of novel stimuli, words presented in a distinctive font, color, and size, and infrequent sounds as compared with a regular “beep” sound. The task utilized in this study was slightly different than the usual von Restorff paradigm. Our learning list contained more than one isolate, resembling the paradigm applied by Kishiyama and colleagues (2004). Like these earlier authors, we replicated the von Restorff effect, which suggests that our manipulation is comparable to other von Restorff paradigms.

Our hypotheses were that these novel stimuli would elicit the novelty complex N2b–P3a. If novelty affects encoding, we would expect that words presented in novel fonts, or coincidentally with novel sounds, would be remembered better, and that the von Restorff effect has a psychophysiological correlate, with higher amplitudes in the N2b–P3a complex for novel words that were correctly recalled later than for those not recalled.

The behavioral data showed, as expected, that the manipulation of the words' size, font, and color was effective in eliciting the von Restorff effect. Words in a novel font were recalled better than standard words. This effect was not present for the recognition task. Actually, novel words tended to be recognized less accurately than standard words. This difference can be explained by the font used in the recognition task: all words were presented in standard font during the recognition test, resulting in a font mismatch for the novel-font words that hurt their recognition. This has also been found previously (Fabiani and Donchin 1995). In cued recall, the cues were also presented in standard font, which may also have led to a mismatch. Although this may have reduced the size of the effect, it clearly did not eliminate the advantage for novel-font words.

As predicted, novel-font words generated a larger N2b–P3a complex: Numerically higher, although not significantly different, amplitudes for the N2b component for novel-font words, localized over frontal sites, higher amplitudes for the P3a component for novel-font words over fronto-central sites, and higher amplitudes for the P3b component for novel-font words over centro-parietal sites. Higher P3 amplitude suggests activation of attention-related regions by novelty (Knight and Scabini 1998).

The exploration made of other components, for the fonts condition, showed enhanced N400 component for novel as compared with standard words. The N400 has been related to detection of significance, and is enhanced when a word in a phrase is discordant to the rest (Chwilla et al. 2007). This suggests that the words presented in different fonts are viewed as somewhat discordant in a semantic sense; if the novelty-related differences were due to just physical features of the words, the N400 component should not differ between novel and standard words. Perhaps there was a stronger processing of meaning for the standard words, than for the novel words, where distinctive fonts and colors might have attracted attention away from the processing of meaning.

For auditory stimuli, ERPs were different than expected. The N2a and P3a components had higher amplitude (positive or negative, accordingly) for the standard than for the novel sounds, while the P3b component had more positive amplitude for the novel than for the standard sounds. This pattern was true regardless of the order of presentation of the sounds after (Experiment 1) or before (Experiment 2) the word. This was unexpected as novel sounds should trigger higher amplitudes on the automatic N2a (Alho et al. 1994) and the semiautomatic P3a components, whereas no difference should be found on the P3b component given that the sounds were task irrelevant (Knight and Scabini 1998; Polich and Criado 2006; Polich 2007). One explanation for these unexpected findings is that the novel sounds resemble familiar ones, as around 80% (35 of 45) of the sounds were either animal or vehicle related, or sounds made by a human voice. However, other studies have found a novelty N2 and P3a using similar sounds (e.g., Kihara et al. 2010).

Another possibility is that the spacing of the sequence of sounds worked against the establishment of the context required for oddball effects: auditory oddball paradigms normally have much shorter interstimulus intervals (Nyman et al. 1990; Kujala et al. 2001; Kihara et al. 2010). Nevertheless, the novel sounds did attract attention of the participants, as indicated by increased P3b amplitudes for novel as compared to standard sounds. A final option is that the complexity difference between the standard “beep” and the novel sounds masked a novelty effect. However, this is not supported by other studies in the field. Ceponiene and his group have found that the differences in the amplitude of the N2 component are opposite to our results, with complex sounds eliciting larger amplitudes than simpler ones (e.g., Ceponiene et al. 2001). In our study, we also found latency differences between complex and simple sounds, with complex sounds having a later latency. Again, this was not found in previous studies. The evidence concerning this matter comes mainly from developmental studies, which have not found any difference in the latency of the N2 component between complex and simple sounds (Ceponiene et al. 2005).

While novel sounds thus attracted attention, words presented with those sounds were recalled less often than words presented with standard sounds. This was true when the sound came during word presentation (Experiment 1), but not if the sound was played before the word (Experiment 2). This suggests that novelty was not aiding encoding; instead, novel sounds attracted attention away from the words when they co-occurred as in Experiment 1, yielding worse memory.

The critical test for the hypothesis that novelty aids encoding is whether we would find a higher N2b–P3a complex for correctly recalled items. In fact, only a main effect was found for the accuracy in the N2b component, but no interaction was found between accuracy and novelty. This indicates that the N2b at acquisition indexes some process that aids later recall. However, this is not novelty processing, as this process is not differentially expressed for novel than for standard-font trials. With respect to the P3a, no difference in amplitude was found between subsequently recalled and not-recalled words. This suggests that the novelty processing indexed by the N2b–P3a is not beneficial for recall. Such a conclusion is consistent with the results from Dunlosky and colleagues (2000). These authors found that isolates presented at the start of a list were remembered better than standard words, even though there was not yet a context to make them distinctive. This finding, and ours, suggests that the von Restorff effect is not produced by novelty processing, but by other mechanisms.

However, there are also studies with findings that contradict ours. Several studies have found that recalled isolates elicited larger P3s at study than nonrecalled isolates (Fabiani et al. 1990; Fabiani and Donchin 1995; Otten and Donchin 2000). This was only the case when color was used to make words distinctive and not when this was done with a surrounding frame (Otten and Donchin 2000), and only when participants were instructed to use rote rehearsal as their learning strategy, not when elaboration was used (Fabiani et al. 1990). Wiswede et al. (2006) found a larger P3 for both recalled isolates and recalled standard words as compared with not-recalled words. In all of these studies, immediate free recall was used to test memory. We tested memory with delayed cued recall and recognition. Our contradictory findings suggest that P3 amplitude indexes a process that helps in free recall, but not cued recall or recognition. One candidate for such a process is attention to the unique, novel feature itself. In free recall, but not cued recall or recognition, features such as color or font size can be used as cue to retrieve the word. For example, in our study only one of 80 words was presented in green font. This would probably make “green” a good cue to retrieve this word in free recall. Thus, in free recall, the “green” word would often be recalled (see McDaniel et al. 2005 for a discussion of similar retrieval-based accounts). By contrast, during cued recall, participants cannot search their memory for a green word, as they would not know for which word stem cue “greenness” would be of any help in retrieval.^2^ If the P3a indeed indexes attention to the novel feature, this would thus aid free recall of isolates (Karis et al. 1984; Fabiani et al. 1990; Fabiani and Donchin 1995; Wiswede et al. 2006), but not cued recall. It would also, presumably, aid more in designs in which there was just a single isolate per list, as opposed to more than one as in our design.

Whether this is the case, it is clear that a von Restorff effect can be found for isolates that do not elicit larger P3s, for example, when isolates are made distinctive with a surrounding frame (Otten and Donchin 2000) or when elaboration is used (Fabiani et al. 1990; our results). Moreover, Fabiani and Donchin (1995) reported larger P3s for recalled as compared to not-recalled isolates in conditions where no von Restorff effect was found: for semantic isolates when participants were asked to focus on physical appearance, and for a recognition task that followed lexical decision. Differences in P3 amplitude between subsequently recalled versus not-recalled words are thus neither sufficient nor necessary for the von Restorff effect. It may therefore be parsimonious to conclude that these P3 amplitude differences are not related to the cause of the von Restorff effect. These causes may lie in easier recall, as suggested by retrieval-based accounts of the effect (e.g., McDaniel et al. 2005), but our results also suggest a role for better learning. Future studies may look more in detail at the processes occurring during retrieval.

If N2–P3 differences are taken as a good indicator of novelty processing, one could further conclude that novelty processing is not the reason for better memory for isolates in a von Restorff paradigm. More speculatively, the role of novelty in learning may be smaller than has been suggested by some (Hasselmo et al. 1996; Meeter et al. 2004; Lisman and Grace 2005). Novelty may mostly be good at attracting attention to itself, and thus away from other material. This may sometimes aid performance, as when a novel feature can be used as a cue to free recall an item. It can also hurt performance, as in our Experiment 1, where novel sounds attracted attention away from the words. Nonetheless, we did find a von Restorff effect in cued recall, which cannot be attributed to use as a cue of novel features. This effect, smaller than in other studies (Otten and Donchin 2000; Wiswede et al. 2006), may be a true effect of novelty on encoding, perhaps through increased rehearsal for novels as has been found in other studies (Dunlosky et al. 2000).

## Conclusions

The von Restorff effect is a robust advantage for isolates within a list. These isolates can generate novelty-associated fronto-central N2 and P3a, and the centro-parietal P3b components. However, this N2–P3 complex is not enhanced for correctly remembered isolates as compared to forgotten ones. This finding, and others, suggest that novelty processing is not the cause of the von Restorff effect, and may not be as advantageous for memory encoding as sometimes thought.

## Notes

1 Further exploration showed a main effect of accuracy over Fz (*F*_1,15_ = 6.70, *P* = 0.02). However, given that this analysis was not planned, this finding cannot be taken at face value – correcting for multiple comparison would not yield a significant result.

2 The exception would be when a cue was presented in the same color/font during cued recall or recognition as during study. In this case, the color/font can be used as cue, and help performance. Indeed, Fabiani and Donchin (1995) found a von Restorff effect in recognition if words were presented during recognition in the same font as during study.
